# Supplementary material for: Pelvic floor disorders and associated factors among women in sub-Saharan Africa: A systematic review and meta-analysis protocol
Source: PLoS One. 2025 Mar 19;20(3):e0319972. doi: 10.1371/journal.pone.0319972 (PMC11922210; doi:10.1371/journal.pone.0319972)
Supplement: S2 Appendix — (DOCX) [file pone.0319972.s002.docx]

Proposed search strategies for **Pelvic floor disorders and associated factors among women in sub-Saharan Africa: A Systematic Review and Meta-Analysis**.

**PubMed**

| **Search** | **Search term** |
| --- | --- |
|  | (“Prevalence” [All Fields] OR (“Magnitude” [All Fields]) |
|  | (“Pelvic floor disorders” [All Fields] OR “genital prolapse” [All Fields] OR “uterine prolapse” [All Fields] OR “pelvic organ prolapse” [All Fields] OR “urinary incontinence” [All Fields] OR “stress urinary incontinence” [All Fields] OR “faecal incontinence” [All Fields] OR “anal incontinence” [All Fields]) |
|  | (“Associated factors” [All Fields] OR “Risk factors” [All Fields] OR “Determinants” [All Fields] OR “Predictors” [All Fields] OR “Correlates” [All Fields]) |
|  | Nigeria OR South Africa OR Ghana OR Tanzania OR Kenya OR Rwanda OR Botswana OR Cameroun OR Senegal OR Angola OR Uganda OR Mali OR Sierra Leone OR Ivory Coast OR Ethiopia OR Lesotho OR Zambia OR Zimbabwe OR Namibia OR Guinea OR Mauritius OR Mozambique OR Niger OR Seychelles OR Burkina Faso OR Burundi OR Cape Verde OR Cameroon OR Central African Republic OR Chad OR Comoros OR Democratic Republic of Congo OR DR Congo OR Djibouti OR Cote D'ivoire OR Congo OR Equatorial Guinea OR Eritrea OR Gabon OR Guinea-Bissau OR Madagascar OR Congo Republic OR Sao Tome and Principe OR Swaziland OR Togo OR Benin OR Liberia OR Namibia OR Gambia OR (Cent Afr Republ) OR (Equat Guinea) OR (Papua N Guinea) OR (Sao Tome E Prin) OR Principe OR Sao Tome and Principe |
|  | Free Full text, observational study, Humans, Women, Female |
|  | 1 AND 2 AND 3 AND 4 AND 5 |

Google Scholar, African Journal Online, EMBASE and CINAHL

“Prevalence OR magnitude AND Pelvic floor disorders OR genital prolapse OR uterine prolapse OR pelvic organ prolapse OR urinary incontinence OR stress urinary incontinence OR faecal incontinence OR anal incontinence AND Associated factors OR Risk factors OR Determinants OR Predictors OR Correlates AND Nigeria OR South Africa OR Ghana OR Tanzania OR Kenya OR Rwanda OR Botswana OR Cameroun OR Senegal OR Angola OR Uganda OR Mali OR Sierra Leone OR Ivory Coast OR Ethiopia OR Lesotho OR Zambia OR Zimbabwe OR Namibia OR Guinea OR Mauritius OR Mozambique OR Niger OR Seychelles OR Burkina Faso OR Burundi OR Cape Verde OR Cameroon OR Central African Republic OR Chad OR Comoros OR Democratic Republic of Congo OR DR Congo OR Djibouti OR Cote D'ivoire OR Congo OR Equatorial Guinea OR Eritrea OR Gabon OR Guinea-Bissau OR Madagascar OR Congo Republic OR Sao Tome and Principe OR Swaziland OR Togo OR Benin OR Liberia OR Namibia OR Gambia OR (Cent Afr Republ) OR (Equat Guinea) OR (Papua N Guinea) OR (Sao Tome E Prin) OR Principe OR Sao Tome and Principe”
