# Supplementary material for: Pelvic floor disorders and associated factors among women in sub-Saharan Africa: A systematic review and meta-analysis protocol
Source: PLoS One. 2025 Mar 19;20(3):e0319972. doi: 10.1371/journal.pone.0319972 (PMC11922210; doi:10.1371/journal.pone.0319972)
Supplement: Attachment — Submitted filename: Response to Reviewers_PFD.docx [file pone.0319972.s004.docx]

**Response to Reviewers**

Dear Editor,

Thank you for the opportunity to submit a revised version of the manuscript “Pelvic floor disorders and associated factors among women in sub-Saharan Africa: A Systematic Review and Meta-Analysis Protocol” for publication in PLOS ONE. We appreciate the time and effort that you and the reviewers dedicated to providing feedback on our manuscript and are grateful for the insightful comments on and valuable improvements to our paper. We have incorporated most of the suggestions made by the reviewers. Those changes appear as tracked changes within the manuscript. Please see below a point-by-point response to the reviewers’ comments and concerns.

**Reviewers’ suggestions and comments to the Authors:**

**Reviewer #1: Reviewer comments:**

The authors present a protocol for a systematic review of pelvic floor disorders and associated factors among women in sub-Saharan Africa. While this is an important topic for understanding the burden and determinants of pelvic floor disorders in this population, the rationale for publishing the protocol, as opposed to the actual study, is not clearly articulated. The protocol outlines a comprehensive approach to data collection and interpretation; however, the authors do not explain why the protocol itself warrants publication. Additionally, the aim and scope of this research might be better addressed through collaboration with the Global Burden of Disease study. Even if the authors wish to pursue this review by analyzing the literature, this approach does not necessarily justify the protocol's publication, as it neither significantly advances the literature nor provides a clear trajectory for future research. Moreover, the authors fail to present a comprehensive search strategy across relevant databases, which is crucial for conducting a systematic review. Given the stated goal of performing a thorough systematic search, it is imperative that the complete strategy be provided.

**Response**: Thank you for your valuable input, and we appreciate your observation. There are several advantages of publishing a review protocol, including informing the academic community about ongoing study and possibly preventing duplication. It also offers transparency and aids in the early detection and resolution of problems. We have uploaded the proposed search strategy as a supporting information, and we can modify it and will upload it along with the final manuscript.

**Reviewer #2: Reviewer comments:**

The review question is clear
The protocol is adequately developed based on international Rw guidelines
Tools such as Covidence helps for an efficient screening of the included studies
The methodological approach using PRISMA-P is adequate and clear as the review protocol.
How the research team will implement each step and who will be involved at each stage is well detailed
Joanna Bridge Institute's (JBI) appraisal should read as Joanna Briggs Institute.

**Response**: Thank you for your encouragement and insightful feedback. Joanna Bridge Institute's (JBI) was typing error and rephrased as Joanna Briggs Institute.

**Reviewer #3: Reviewer comments:**

Thank you for affording me the valuable opportunity to review this manuscript.
This methodological study offers significant insights into the prevalence and risk factors of pelvic floor disorders (PFDs) in sub-Saharan Africa, an area where existing evidence remains limited. The findings are poised to make a substantial contribution to the field.
However, the inclusion criteria need to be reassessed.
Pelvic floor disorders (PFDs) can be evaluated through clinical examination or self-administered questionnaires. For instance, the Pelvic Floor Disability Index (PFDI-20) is a self-reported questionnaire that does not determine prevalence but quantifies symptom burden.
In such cases, the findings primarily elucidate symptomatic presentation rather than true prevalence, as exemplified in the reference study (Suemitsu T, Mikuni K, Matsui H, Suzuki M, Takahashi T. Prevalence and Risk Factors of Pelvic Floor Disorders After Delivery in Japanese Women Using the Pelvic Floor Distress Inventory: A Retrospective Cohort Study. Cureus. 2023;15(6):e40152. Published 2023 Jun 8. doi:10.7759/cureus.40152).
The inclusion criteria will inevitably vary depending on whether the study aims to determine prevalence or symptom burden. Conducting a preliminary evaluation of previous studies to clarify their assessment methods, the volume of relevant literature, and the reliability of these sources would be prudent.

Additionally, I suggest considering treatment and intervention strategies, including rehabilitation, as secondary evaluation outcomes. Identifying risk factors does not always equate to their mitigation. For example, the increased incidence of PFDs associated with instrumental delivery, obstetric anal sphincter injuries (OASIS), and epidural analgesia is a well-documented phenomenon worldwide, not exclusive to sub-Saharan Africa.
A review of this nature may yield findings similar to previous studies. Nevertheless, presenting potential solutions would substantially enhance the review's significance. Therefore, I recommend incorporating this aspect into the evaluation criteria.

**Response**: Thank you for pointing this out. We have modified our inclusion criteria to “Observational studies that reported the prevalence of women with PFDs using validated data collection tools and conducted in sub-Saharan Africa …”. We have carried out a preliminary study to identify how studies defined PFD (whether through clinical examination or self-report). All of the studies we observed used one of the validated data collection tools, including the Pelvic Floor Distress Inventory Short Form (PFDI-20), Epidemiology of Prolapse and Incontinence Questionnaire (EPIQ), and the International Consultation on Incontinence Questionnaire (ICIQ). We will forward recommendations to stakeholders and interested organizations based on the review’s findings to contribute to the development of suitable care and preventative strategies.

**Reviewer #4: Reviewer comments:**

I would like to thank you for the opportunity to review the study protocol entitled "Pelvic Floor Disorders and Associated Factors Among Women in Sub-Saharan Africa: A Systematic Review and Meta-Analysis Protocol". This protocol raises considerations regarding the inclusion of studies for analysis, and I would like to make a few suggestions.

First, it is important to determine whether the studies selected for review have adequately addressed the prevalence of pelvic floor disorders. It is recommended that the included studies use validated assessment tools that are appropriate for the target population. The lack of validity may be a source of bias in the results. Some examples of validated instruments are the Epidemiology of Prolapse and Incontinence Questionnaire (EPIQ), the Pelvic Floor Distress Inventory Short Form (PFDI-20), and the International Consultation on Incontinence Questionnaire (ICIQ), etc.

In addition, it is important to clarify whether the approach will rely solely on clinical history to identify pelvic floor dysfunction, or whether both methods will be used. It is important to include validated methods in the data extraction, as this would provide a more robust basis for the results obtained. Therefore, it would be beneficial to include a specific field in the Excel file for data extraction to indicate how prevalence was measured in each study.

Finally, it is important to consider whether pelvic pain should be included or excluded from the analysis alongside pelvic floor dysfunction for a global analysis of pelvic floor dysfunction.

**Response**: we are grateful for your valuable feedback. Pelvic floor disorders (PFDs) can be evaluated through clinical examination or validated questionnaires. We will consider any study that can report the prevalence of women with PFDs using validated data collection tools and conducted in the SSA, though we have not identified a study that used clinical examination to decide PFD during our preliminary study. pelvic pain will not be assessed in the current study as an outcome variable.
